# The ins and outs of microglial cells in brain health and disease

**DOI:** 10.3389/fimmu.2024.1305087

**Published:** 2024-04-11

**Authors:** Carla Pallarés-Moratalla, Gabriele Bergers

**Affiliations:** VIB-Center for Cancer Biology, KU Leuven, Leuven, Belgium

**Keywords:** microglia, neuroinflammation, depletion, repopulation, re-education, stroke, aneurysm, glioblastoma

## Abstract

Microglia are the brain’s resident macrophages that play pivotal roles in immune surveillance and maintaining homeostasis of the Central Nervous System (CNS). Microglia are functionally implicated in various cerebrovascular diseases, including stroke, aneurysm, and tumorigenesis as they regulate neuroinflammatory responses and tissue repair processes. Here, we review the manifold functions of microglia in the brain under physiological and pathological conditions, primarily focusing on the implication of microglia in glioma propagation and progression. We further review the current status of therapies targeting microglial cells, including their re-education, depletion, and re-population approaches as therapeutic options to improve patient outcomes for various neurological and neuroinflammatory disorders, including cancer.

## 1 Introduction

The brain is a highly specialized and complex organ that controls vital functions ranging from cognitive processing, motor skills, vision, generation and regulation of emotions, and body homeostasis. Most regions of the brain are shielded in the form of a blood-brain barrier (BBB) against potentially harmful substances to maintain a stable and tightly regulated microenvironment essential for optimal brain function, (1, 2).

The brain harbors a complex network of cell types, including neurons, glial cells, innate immune cells, and mesenchymal cells, each contributing to specialized functions (3). While neurons facilitate signal transduction and communication within the brain and throughout the body by enabling sensory, motor, and cognitive functions (4), glial cell types play a crucial role in supporting and protecting the brain’s structure and functionality. Astrocytes are the most abundant glial cells, which regulate cerebral blood flow, the blood-brain barrier, and tissue repair following infection and traumatic injuries (5). Oligodendrocytes produce myelin sheaths for the protective insulation of nerve cells, facilitating rapid signal transmission (6). Brain-resident macrophages entail perivascular, meningeal, and choroid plexus macrophages that are localized at the interface between the parenchyma and the circulation (7), and microglial cells which are distributed throughout the entire brain and serve as immune sentinels, actively surveilling the brain microenvironment, participating in both innate and adaptive immune responses, and protecting the brain from potential threats such as injury and infection (8–10). Mesenchymal cells include endothelial cells, pericytes, and smooth muscle cells that form a widespread and extensive vascular network to provide nutrients and oxygen, remove waste products, and support the maintenance of neighboring cells. The intricate interplay between blood vessels, astrocytes, and microglial cells ensures the proper function of the blood-brain-barrier, contributing to overall neurological health (8, 9, 11).

In this review, we will focus on microglial cells and discuss their responsibilities under physiological conditions and their impact on cerebrovascular diseases (i.e., stroke, aneurysm, and glioma), taking a recent perspective of microglial experts into consideration that proposes a revised nomenclature to display the dynamic and multilayered states of microglial cells. We explore 1) microglia functions in health and disease, 2) the differences between brain-resident macrophages and monocyte-derived infiltrating macrophages, 3) the mechanisms of microglia re-education, depletion, and repopulation, and 4) highlight new insights into myeloid adoptive cell transfer as a new therapeutic approach for cerebrovascular diseases. This will provide a better understanding of how microglial cells modify brain homeostasis upon injury and pave the way to improve the efficacy of current therapies for stroke, aneurysm, and glioma by targeting the brain resident macrophages.

## 2 Microglial cells

### 2.1 Microglia origin and function

**Figure 1:**
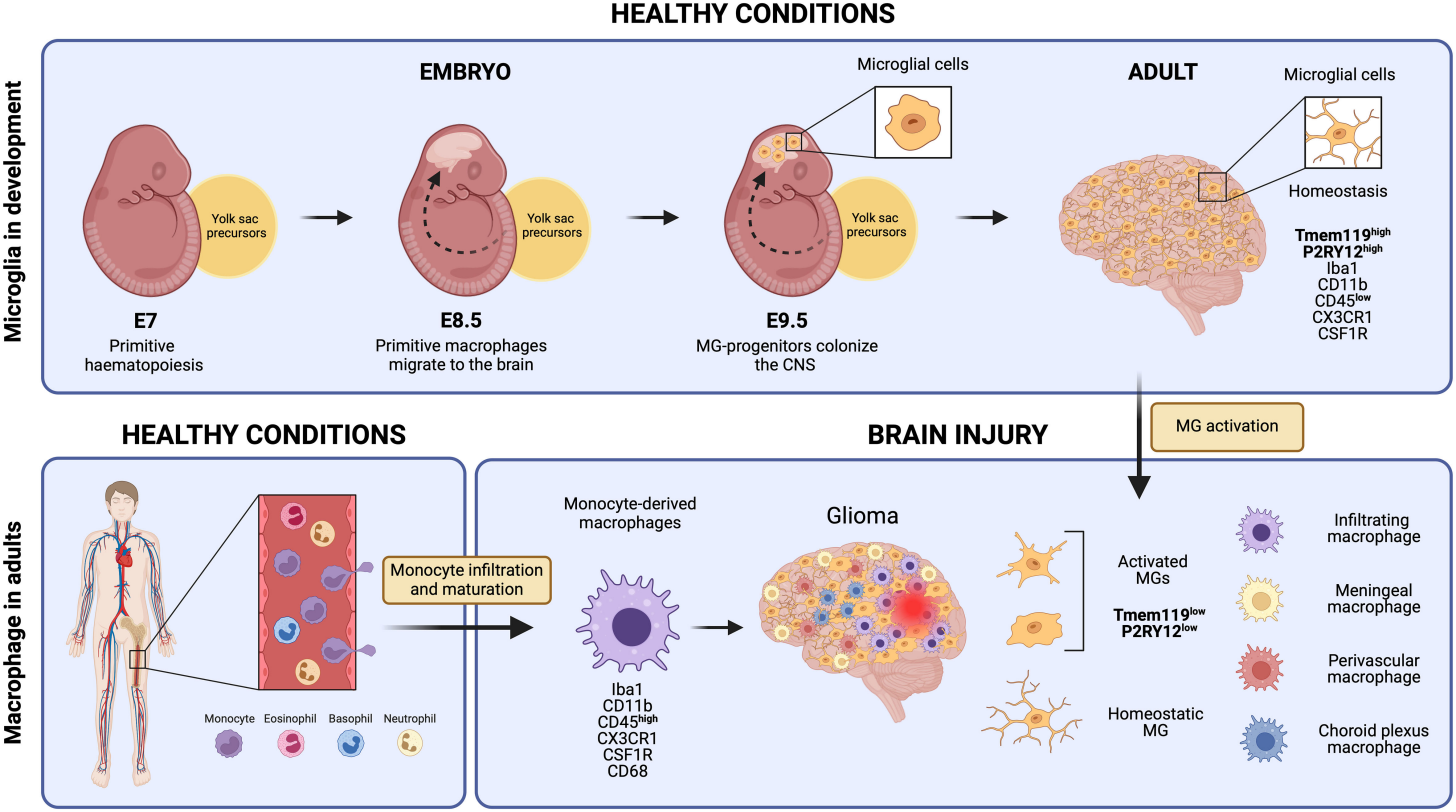
Microglial cells originate from yolk sac precursors around day 9.5 of embryonic development and populate the entire Central Nervous System. Under normal, healthy conditions, microglia maintain a homeostatic state expressing unique markers that set them apart from monocyte-derived macrophages, such as Tmem119 and P2RY12. However, in response to brain injury, microglial cells undergo activation and subsequently down-regulate some of these specific markers. This downregulation makes it extremely challenging to distinguish them from monocyte-derived macrophages that reach the injury site through monocyte infiltration and maturation, as well as other brain-resident macrophages like meningeal macrophages, perivascular macrophages, and choroid plexus macrophages. Created by Biorender.

Microglial cells derive from primitive macrophages that originated in the yolk sac during early embryonic development, setting them apart transcriptionally from other tissue-resident macrophages and bone marrow-derived macrophages (**Figure 1**) (10, 12). In mice, primitive hematopoiesis occurs in the yolk sac around day seven (E.7) of embryonic development and contributes to producing the first erythrocytes and macrophages. Primitive macrophages appear in the blood islands of the yolk sac around day nine of embryonic development (E.9). These yolk sac-derived primitive macrophages migrate to different tissues, including the brain, through the blood after the establishment of the circulatory system (from E8.5 to E10) where they differentiate into fetal macrophage populations (13). They enter the brain at E9.5, before the BBB formation, and colonize the brain parenchyma via migration and proliferation mechanisms (14, 15). Microglia proliferate in response to colony-stimulating factor 1 (CSF-1), granulocyte-macrophage colony-stimulating factor, interleukin 4 (IL-4), and interleukin 5 (IL-5) (16). They reach their highest population two weeks after birth and are maintained until adulthood (17, 18). While microglia can self-renew to sustain their population, other fetal macrophage populations are replaced by fetal liver-derived monocytes that colonize the tissue later and differentiate into macrophages (19). The latter is conceivable with the observation that the generation of the BBB in rodents coincides with the release of fetal liver monocytes into the blood circulation at about E13.5. Yolk sac-derived primitive macrophages, however, start to invade the brain tissues earlier at E9.5 (20) (**Figure 1**).

Different mechanisms have been proposed by which microglial distribution may occur in the developing brain parenchyma. Microglial cells could migrate along developing blood vessels because migration happens at the same time as vascularization and blood circulation (E10), and vascular sprouts can modulate microglial migration during mouse embryonic development (14, 21). In addition, microglial migration could also be influenced by the modulation of the extracellular matrix by matrix metalloproteinases (MMPs) as inhibition of MMP-8 and MMP-9 impaired microglial spreading (22). Other studies demonstrated that microglia in the postnatal Central Nervous System (CNS) can migrate along the vasculature via the C-X3-C motif ligand 1 (CX3CL1)/C-X3-C receptor 1 (CX3CR1) axis (23). In support, microglial CX3CR1 knockout delayed the microglial recruitment in the early postnatal hippocampus and somatosensory cortex by several days (24).

Once microglia have colonized the brain parenchyma, they will undergo differentiation, which relies on environmental cues. Thereby, microglia require signaling through the colony-stimulating factor 1 receptor (CSF1R) by macrophage colony-stimulating factor 1 (CSF-1) produced primarily by oligodendrocytes and astrocytes for their survival, differentiation, and maintenance as underscored by the observation that CSF1R knockout mice are severely diminished in the number of microglial cells (17, 25). In addition, interleukin 34 (IL-34), another CSF1R ligand, secreted by neurons, and the hematopoietic transcription factor PU.1 can also contribute to microglial homeostasis and development, respectively (22, 26). Interestingly, transforming growth factor β (TGF-β) and interleukin-33 (IL-33) secreted by mature neurons and astrocytes, respectively, were shown to trigger post-natal microglial differentiation (27, 28). In addition, a recent study indicated that brain-resident CD4^+^ T-cells (CD69^+^) play a key role in the post-natal transcriptional and morphological maturation of microglia, both in mice and humans. The absence of murine CD4^+^ T-cells impaired microglial maturation to the final adult state, resulting in immature neuronal synapsis, defective synaptic pruning, and behavioral abnormalities (29).

Throughout adulthood, microglia undergo turnover approximately every four years. This means that over a lifetime, they will be renewed several times, primarily through self-renewal mechanisms among existing microglia (30, 31). Interestingly, studies have demonstrated that peripherally derived macrophages can replace depleted microglia in certain situations. However, it is essential to note that these replacement cells retain their unique identity and differ from microglial cells (32).

### 2.2 Microglia communication with other cell types in the brain environment

**Figure 2:**
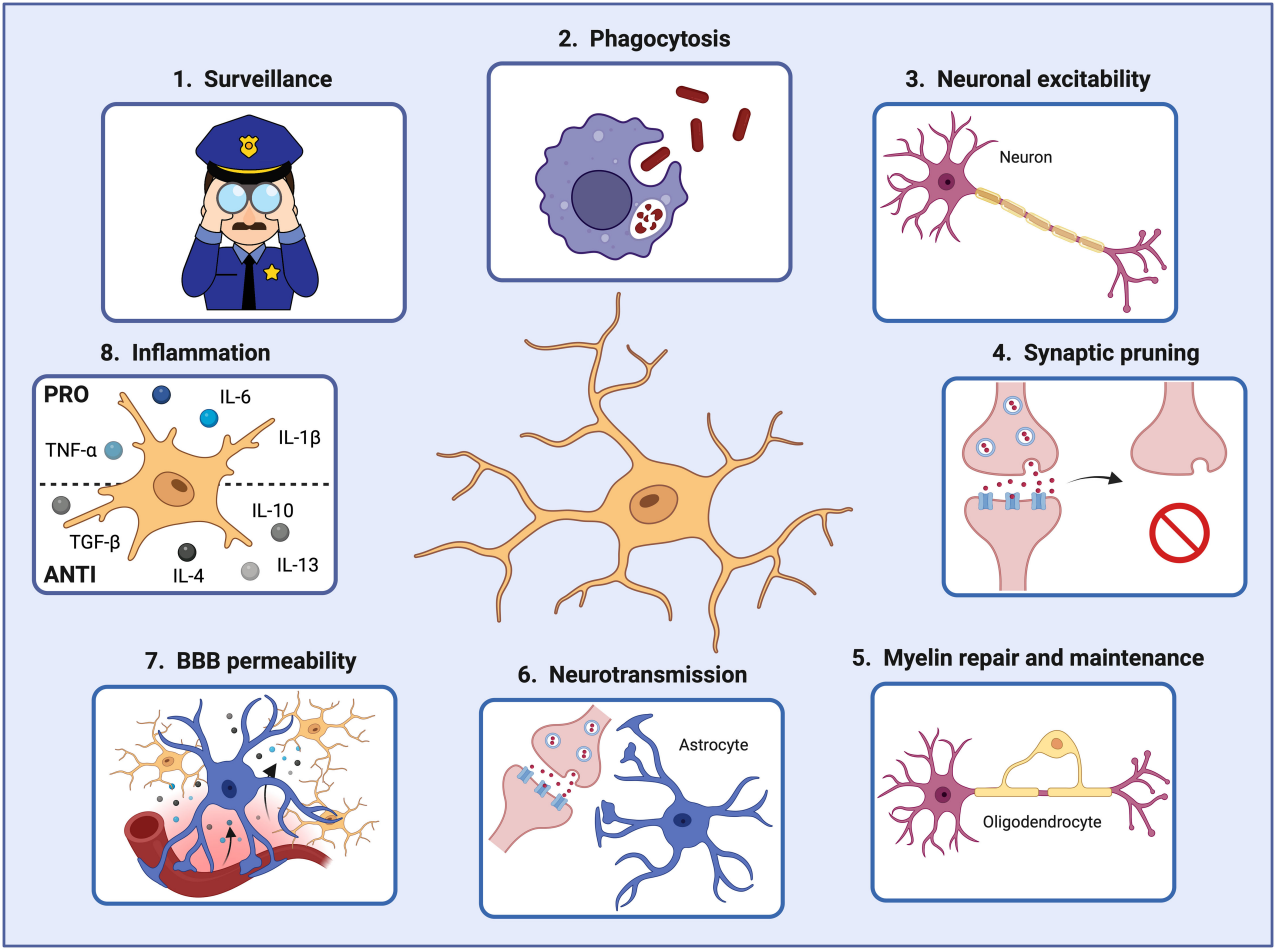
Microglial cells play crucial roles in the brain during development and adulthood. In order to maintain brain homeostasis, microglial cells 1) act as sentinels constantly surveilling the brain environment and adapting their functions to specific triggers, 2) phagocytose pathogens and damaged cells that may threaten the brain homeostasis and function, 3) regulate neuronal excitability, 4) prune defective synapses, 5) interact with oligodendrocytes for myelin repair and maintenance, 6) assist astrocytes in controlling neurotransmitter levels, 7) modify the blood-brain barrier permeability by secreting factors that increase the expression of cell adhesion molecules in endothelial cells, and 8) secrete pro- and anti-inflammatory factors controlling the inflammatory response. Created by Biorender.

Under steady-state conditions, MGs actively interact with various brain cell types, including neurons, astrocytes, oligodendrocytes, pericytes, and endothelial cells, to maintain a functional and healthy brain (33–35). For instance, MGs support neuronal activity in different ways. Firstly, MGs can prune defective synapses and regulate neuronal excitability (36, 37). They can clear debris produced by dying neurons and modify the synapsis by partial phagocytosis (trogocytosis) and by interposing themselves between pre- and post-synaptic membranes (synaptic stripping) (38–40). Secondly, they can also influence neuron proliferation and migration and promote their survival by secreting growth factors such as insulin-like growth factor-1 (IGF-1), essential for protection against cytokine-induced neuronal cell death (41) (**Figure 2**).

In addition, microglia can interact with astrocytes and help to control the brain’s neurotransmitter levels. By releasing small amounts of ATP, astrocytes are recruited, and glutamate is released. Astrocyte-secreted glutamate increases the excitatory postsynaptic currents via neuronal metabotropic glutamate receptor 5 (mGluR5), improving the synaptic transmission and keeping the brain homeostasis (42). Moreover, microglia also interact with oligodendrocytes, the cells responsible for myelin production. A recent study demonstrated that microglia depletion in adult mice resulted in enlarged inner tongues and ticker myelin (43). Another study discovered that microglia help repair demyelinated lesions via post-squalene sterol synthesis (44). Thus, microglia are essential in helping oligodendrocytes maintain and repair the myelin for optimal neuronal communication and overall brain function (33) (**Figure 2**).

Furthermore, microglia, being macrophages, communicate with endothelial cells. Microglia can secrete factors such as TNF-α and IL-1β that increase the expression of leukocyte adhesion molecules (i.e., ICAM-1 and VCAM- 1) on vascular endothelial cells, promoting the infiltration of peripheral immune cells into the brain parenchyma (11, 34). Thereby, microglia can also interact with the infiltrating immune cells via MHC-II-like molecules, activating T-cells to become pro-inflammatory and consequently releasing inflammatory factors that will initiate and regulate the immune response within the brain during pathological conditions (45, 46) (**Figure 2**).

In conclusion, microglial cells interact with other cell constituents in the brain, like neurons, astrocytes, oligodendrocytes, endothelial cells, and immune cells, via different mechanisms aiming at protecting the immune-privileged brain and maintaining homeostasis.

### 2.3 Microglial cell states

Recently, a consortium of experts in the field has put forward a revised view of the dynamic and complex microglia states in relationship to their specific functions in development, homeostasis, aging, and disease and provided recommendations on the use of a revised microglial terminology that is more appropriately reflective of their complex and dynamic states (47). In line with their proposition, microglia should be considered highly dynamic and plastic cells that display co-existing and interchangeable phenotypes in response to environmental cues like “wearing several hats which they can continuously exchange”. There is a common census that the historical and still current view of dividing microglia function into dichotomic categories (e.g., M1 versus M2, resting versus activated) is prone to misconceptions, while a multidimensional integration of data on their transcriptomics, metabolomics, morphology, location, or epigenetics will help to reveal a more complete picture of their nature and function.

For example, microglia have been commonly described to be in a “resting state” during homeostatic conditions as characterized by their ramified morphology and modest local proliferation. However, homeostatic microglial cells are anything but resting as they continuously patrol the brain environment and promptly react when they detect the first signs of infection or tissue damage. While microglia respond locally to minor tissue insults detected, they react much more rigorously upon severe injury or formation of neuroinflammatory and neurodegenerative disturbances. This is commonly accompanied by morphological changes, sometimes adopting an amoeboid morphology with rapid clonal expansion throughout the brain, making it challenging to distinguish microglia from bone marrow-derived macrophages (**Figure 1**) or other monocytes that are recruited to the injury site and elicit immune responses (48).

Upon activation by injury or inflammation, microglia, like macrophages, set out to eliminate any threats first but then subsequently support tissue repair and wound healing. Due to their phagocytic capability, they are instrumental in removing cell debris and foreign microorganisms. They are also involved in synaptic pruning, transmission, and regulation of neuronal excitability, as well as astrocyte activation (12, 49) (**Figure 2**).

In the context of injury, microglia exposed to microbial products or damaged tissue, release pro-inflammatory molecules such as interleukin-1 β (IL-1β), interleukin-6 (IL-6), tumor necrosis factor-alpha (TNF-α), and reactive oxygen species (ROS) that help initiate and amplify inflammatory responses, recruiting and activating immune cells (i.e., T and B cells) and promoting phagocytosis of pathogens or damaged cells. In contrast, during tissue repair and regeneration, microglia secrete anti-inflammatory cytokines, such as interleukin 10 (IL-10), transforming growth factor β (TGF-β), interleukin 4 (IL-4), and interleukin 13 (IL-13) to abrogate inflammation and promote wound healing (50, 51) (**Figure 2**). In addition, a recent study demonstrated the pivotal role of microglia in neocortex inflammation since they produce and secrete CCL2, a chemokine involved in immune cell recruitment and BBB-microvessel leakage. Notably, decreased MG-CCL2 expression was associated with a more restored BBB and diminished neuroinflammation (52). It is important to note that the immune status, referred to as polarization of microglia or macrophages, will be determined by the net effect of a variety of pro-inflammatory and anti-inflammatory stimuli, being the outcome of the response a balance between the strength of the detected signals. This fine-tuned process is influenced by complex interactions with neighboring cells, including neurons, astrocytes, and endothelial cells, as well as by specific signaling molecules (50, 53).

Changes in the activation state of microglial cells, and macrophages in general, also require metabolic rewiring. Microglia display high glycolytic activity and glutaminolysis to meet their energy demands for surveillance, motility, and immune responses (54, 55). Pro-inflammatory microglia obtain their ATP energy via aerobic glycolysis by increasing their glucose uptake and lactate production (56). In addition, they activate their pentose phosphate pathway (PPP) to support DNA replication and RNA transcription (56, 57). In contrast, immunosuppressive/anti-inflammatory microglia/macrophages use mostly mitochondrial oxidative phosphorylation for ATP production (56, 58) and increase fatty acid oxidation to fulfill their energy demands (56, 59). The ability of microglia to use different energy sources enables them to promptly adapt to variable metabolic conditions. Metabolic rewiring eases their response by adapting quickly to a changing microenvironment in the healthy brain and in disease.

Transcriptional profiling has helped to identify specific gene signatures that are distinct between microglia and monocyte/macrophage populations. The genes *P2ry13*, *P2ry12*, *Slc2a5*, *Sall1*, *Olfml3*, and *Tmem119* are highly expressed in microglial cells, while monocytes and macrophages exhibit higher levels of *F10*, *Emilin2*, F5, *Gda*, *Mki67*, *Sell*, and *Hp* (60). Both macrophage cell types share IBA-1, CX3CR1, F4/80, CD11b, CD45, and CD68 expression. So far, transmembrane protein 119 (TMEM119) and purinergic receptor P2Y12R are the most specific and broadly used markers for (homeostatic) microglia but activated microglia tend to downregulate their expression (61–63) (**Figure 1**). Using flow cytometry, microglial cells can be distinguished from bone marrow-derived macrophages by differential expression of the CD11b, CD45, Lys6C, and Lys6G markers because microglia are CD11b^+^ CD45^low^ Lys6C^-^ Lys6G^-^ while infiltrating monocytes/macrophages are CD11b^+^ CD45^high^ Lys6C^low^ Lys6G^-^ (64). This distinction, however, becomes challenging during inflammation and aging in which microglia can upregulate the expression of these markers (i.e., CD45) (60, 65). In summary, microglia quickly adapt to their environment and modify their functions with a spectrum of activation states which is reflected in their distinct expression profiles.

### 2.4 Understanding microglia plasticity by inducing microglia re-population

Microglial cells play a crucial role in maintaining the delicate balance of the brain’s microenvironment. Their essential functions and distinct origin from other infiltrating macrophages have drawn attention to understanding the consequences of depleting microglia in the brain. Microglia depletion has provided insight into the ability of the brain to repopulate microglia from different cell resources, underscoring their essential functions. These studies also exposed the extent of the repopulating cell constituents in immune surveillance and neural-supporting functions during brain homeostasis.

### 2.5 Microglia depletion in the brain

**Figure 3:**
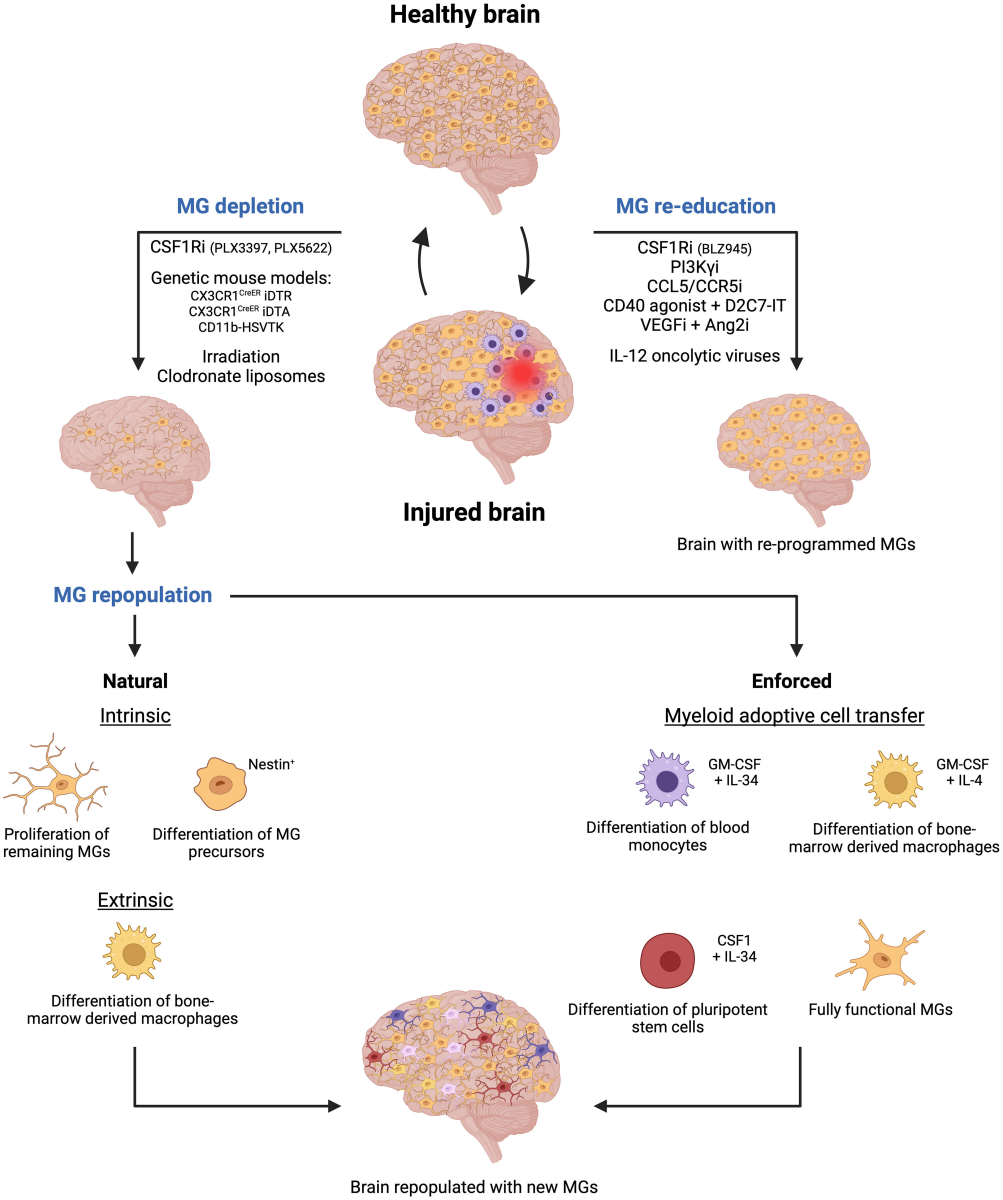
Microglia-targeted therapies are emerging as a novel therapeutic approach for cerebrovascular diseases. Two primary strategies are being explored to target microglial cells following brain injury. The first strategy involves depleting microglial cells using methods such as pharmaceutical inhibitors or genetic approaches. Subsequently, new microglial cells will repopulate the empty niche through natural processes like proliferation of remaining microglial cells, differentiation of nestin^+^ microglia precursors, and differentiation of bone marrow-derived macrophages. However, enforced repopulation can occur through myeloid adoptive cell transfer leading to the emergence of new microglia from the differentiation of blood monocytes, bone marrow-derived macrophages, and pluripotent stem cells, or upon direct transfer of fully functional and activated microglial cells. The second strategy focuses on re-educating existing microglial cells using specific inhibitors or oncolytic viruses, thereby activating them to contribute to maintaining brain homeostasis following injury. Created by Biorender.

To date, several methods, using pharmacological and genetic approaches have been used to deplete microglial cells by abrogating crucial signaling cues. For instance, colony-stimulating factor 1 receptor (CSF1R) inhibitors (i.e., PLX5622, PLX3397 (Plexxikon), BLZ945 (Novartis)) that pass the BBB, have been extensively used to deplete microglia in several animal models (66–68). These inhibitors block the CSF1R activation, which is essential for microglial differentiation and survival. However, it is important to note that blocking CSF1R signaling will affect both infiltrating monocyte-derived macrophages and resident microglial cells (69) (**Figure 3**).

In addition, cell-specific genetic strategies have been used to either genetically delete the gene of interest whose products are crucial for microglial survival or use a cell-type-specific approach to kill microglial cells directly. For example, the Cre-loxP technology has been utilized to drive the expression of the diphtheria toxin receptor (DTR) in microglia cells in CX3CR1^CreER^ iDTR transgenic mice. Thus, when the diphtheria toxin is administered upon tamoxifen administration in mice, it selectively targets and kills the DTR-expressing microglial cells (70, 71). Similarly, microglia were depleted through the administration of tamoxifen, which induced the expression of the toxic DTA (Diphtheria Toxin A subunit) protein in CX3CR1^CreER^ DTA transgenic mice (69, 72) (**Figure 3**).

Another approach is the depletion of microglial cells in the CD11b-HSVTK transgenic model. In this mouse model, the herpes simplex virus thymidine kinase (HSVTK) gene is under the control of the CD11b gene promoter, a gene for integrin alpha that is expressed in macrophages and microglial cells. HSVTK is an enzyme that converts the antiviral drug ganciclovir (GCV) into a toxic form. Thus, upon ganciclovir administration to the mice, HSVTK converts GCV into a toxic compound in CD11b^+^ cells. As a result, microglia and infiltrating macrophages are depleted in the brain (69, 73) (**Figure 3**).

Additionally, other alternative methods have been used to deplete microglial cells. In certain experimental settings, irradiation has been shown to deplete microglia, since it affects rapidly dividing cells, like activated microglia, leading to their depletion. This method is often combined with bone marrow transplantation to replace the depleted microglia with microglia, derived from donor-derived bone marrow cells (74, 75). Moreover, liposome encapsulating clodronate, a drug that induces cell death in phagocytes, has also been used to deplete MGs. When these liposomes are administered to mice, microglial cells, but also other myeloid cell types including neutrophils, internalize clodronate and undergo apoptosis (76, 77) (**Figure 3**).

Importantly, circulating monocytes and peripheral tissue macrophages can also be affected upon microglial depletion as genetic and pharmacological approaches cannot distinguish between these populations. Only when pharmacological inhibitors were directly delivered to the brain, were circulating monocytes and peripheral tissue macrophages not reduced. Thus, specific delivery strategies should be taken into consideration for microglia depletion and replacement therapies (78).

Nevertheless, recent research has identified promising potential markers, such as Tmem119 and P2RY12, which are uniquely expressed in resident microglia (61–63). Notably, Tmem119-CreER and P2ry12-CreER mice have already been generated to manipulate gene expression in brain-resident microglial cells without altering monocyte-derived macrophages (79, 80).

### 2.6 Microglia repopulation in the brain

The depletion of microglia can be very efficient, but microglial repopulation is quickly stimulated in the brain, restoring the CNS niche in about 1-2 weeks, specifically when the depleting agent is removed (81).

In detail, microglial cells can repopulate the brain in three different ways: 1) the residual microglia can self-renew by increasing their proliferative capabilities and repopulating the empty niche (81, 82), 2) nestin^+^ microglial progenitors can differentiate into mature microglia replenishing the brain (69, 83), and 3) bone marrow-derived macrophages can infiltrate the brain and acquire a “microglia-like” identity with a distinct transcriptomic profile compared to the original resident microglia (32, 69, 73) (**Figure 3**). The latter restoration process is also very powerful in other organs. For example, circulating monocytes can fully replace Kupffer cells in the liver and alveolar macrophages in the lung with nearly identical cellular phenotypes (84, 85).

Importantly, depending on the method of depletion, the repopulation sources may be different. For instance, it has been shown that microglia depletion with the CSF1R-inhibitor PLX5622 leads to repopulation by which remaining resident microglia increase proliferation (86). Similarly, MG-depletion using the CX3CR1^CreER^ iDTR mouse model induced repopulation by self-renewal of residual microglia (87). However, genetic ablation using the CX3CR1^CreER^ DTA mouse model induced MG-repopulation via both differentiation of monocyte-derived macrophages and proliferation of local microglia (88). Interestingly, the depletion of microglial cells using the CD11b-HSVTK transgenic model promoted the infiltration and differentiation of monocyte-derived macrophages to replenish microglia in the brain (73).

In addition, repopulation of microglia upon depletion may happen at different time points depending on the depletion methods, previously used. For instance, administration of liposome-encapsulating clodronate leads to microglia depletion on day 1, lasting the effects for 3 days, and promoting MG-repopulation 5 days after treatment (76). However, in the CX3CR1^CreER^ iDTR mouse model, most of the MGs are depleted after 3 days upon diphtheria toxin administration, and their repopulation occurred later on day 14 (87). Similarly, in the CD11b-HSVTK mouse model, MG-repopulation happens after 2 weeks (73). Furthermore, CSF1R inhibition using PLX5622 depleted microglial cells after 1-2 weeks of administration and promoted their repopulation 2 weeks after the treatment stopped (89).

These observations highlight the different mechanisms of microglia repopulation upon their depletion in the brain. Understanding the timing and modes of repopulation is crucial for anticipating the consequences of microglia targeting, not only in the context of brain homeostasis but also in the context of brain diseases as detailed in the following chapters. It is pivotal to characterize the repopulated microglial cell population in response to the depletion method. This knowledge is essential for identifying the origin and function of the newly replenished microglia, which may likely display some differences in their transcriptomic profiles and functions compared to the original microglial population.

## 3 Microglia in cerebrovascular diseases

### 3.1 Stroke, aneurysm, glioma

Given microglia’s functions in tissue repair, it is not surprising that they are involved in shaping the outcome of cerebrovascular diseases such as stroke, aneurysms, and gliomas. (90–92). However, their responses to these CNS insults can exacerbate or reduce disease progression depending on their conversion into specific states, as recognized by specific molecular signatures. For example, disease-associated microglia (DAM), which are found at sites of neurodegeneration, have protective effects. They are endowed with a sensory mechanism that involves the Trem2 signaling pathway, to detect damage within the CNS in the form of neurodegeneration-associated molecular patterns (NAMPs). NAMPs are danger signals present on apoptotic bodies of dying neural cells, myelin debris, lipid degradation products, and extracellular protein aggregates typical for neurodegenerative diseases. NAMP binding to microglia triggers their transition into DAMs, whose primary function is to contain and remove the damage (93). Notwithstanding, there is also increasing evidence that microglia actively contribute to the initiation and progression of CNS disorders, including Alzheimer’s disease, amyotrophic lateral sclerosis, and brain tumors (94–99).

### 3.2 Microglial cell behavior in stroke

Stroke is a common cerebrovascular disease characterized by interrupting the blood flow to the brain, leading to oxygen and nutrient deprivation. As a result, events like inflammation, oxidative stress, and cell death occur in the affected areas. There are two main types of strokes: 1) ischemic stroke, the most common stroke caused by a blockage of an artery or a vein interrupting the blood flow to the brain, and 2) hemorrhagic stroke, caused by bleeding into or around the brain. Ischemic stroke is the most common type of stroke since it accounts for around 70-80% of all stroke cases, while hemorrhagic stroke accounts for around 10-20% (2). Both stroke types will promote BBB disruption, neuronal death, and neuroinflammation, which will lead to severe neurological problems (1, 3). So far, treatments for both kinds of strokes are minimal, urging the need to identify therapies that decrease the chances of brain injury and improve recovery upon stroke (4).

After stroke injury, microglial cells can be rapidly activated and migrate into the damaged areas to regulate brain recovery (8). Depending on the activation signals they receive, they can exert dual roles, either promoting injury or facilitating repair (90). In the acute phase of ischemic stroke, microglia/macrophages produce anti-inflammatory cytokines like IL-10, protecting neurons against oxygen and glucose deprivation while promoting tissue repair and regeneration. However, after the acute phase, microglia/macrophages contribute to the exacerbation of inflammation and promote cell death expressing cytokines such as IL-6 and TNF-α (100, 101). In addition, the depletion of microglia 24 hours after an ischemic stroke worsened the ischemic lesion, most probably due to an increased inflammatory response of the remaining macrophages.

Interestingly, in a hemorrhagic stroke, microglia/macrophages experience a distinct phenotypic switch from an immune-activating phenotype during early/acute hemorrhagic stroke towards an immuno-suppressive phenotype in later stages (within 3-7 days after injury) (102, 103).

### 3.3 Microglial cell behavior in brain aneurysm

Cerebral aneurysms are abnormal swellings or bulges in the blood vessels of the brain (91, 92). There are four main types of intracranial aneurysms: saccular, fusiform, mycotic, and dissecting. Saccular aneurysms are the most common type, accounting for around 90% of all aneurysm cases. They are formed by a blood-filled sac that protrudes from the main artery or one of its branches (104).

A vessel leak or rupture by an aneurysm leads to bleeding into the surrounding tissue in the brain, causing a hemorrhagic stroke, also called a subarachnoid hemorrhage. Studies in animals with subarachnoid hemorrhage have shown that microglial cells undergo dynamic changes transitioning from proinflammatory to anti-inflammatory states (105, 106). Congruently, several clinical studies have also provided evidence that microglial cells accumulate and respond to subarachnoid hemorrhage (107, 108). Thereby, microglial cells release pro-inflammatory mediators like IL-6 and TNF-α that may contribute to secondary brain injuries, neuronal cell death, increased blood-brain barrier permeability and vascular damage, and recruitment of perivascular macrophages, perpetuating the cycle of injury and inflammation (91, 109). However, erythropoietin, a hormone, that regulates red blood cell production, can induce a shift in microglial polarization toward an immunosuppressive phenotype (91, 110). Thereby, microglial cells secrete the anti-inflammatory cytokines and chemokines IL-4 and IL-17 (111), upregulate glutamate receptor 5 (GLUT5) and reduce the production of pro-inflammatory cytokines (i.e. IL-1β, IL-6, TNF-α) (112). This decreases neuronal cell death and clears cell debris, altogether facilitating the resolution of cerebral injury and hemorrhage resulting in a neuroprotective effect (91, 110).

### 3.4 Microglial cell behavior in glioma

Gliomas are the most common malignant brain tumors in the CNS. Among the different types of gliomas, glioblastoma multiforme (GBM) is the most prevalent and deadly primary brain tumor in human adults. GBMs entail several features of aggressive growth, including a high proliferative rate, resistance to apoptosis, diffuse infiltration and invasion, propensity for necrosis and hypoxia, and vigorous and abnormal angiogenesis (113, 114). In addition, the capacity of GBMs and lower-grade gliomas to invade and infiltrate normal brain tissues makes their complete surgical removal impossible. The residual tumor remnants and their paucity of standard and targeted therapies provide GBM patients with a poor prognosis and a mean survival time of 15 months only (115–117). Thus, the most important survival predictor is still the extent of surgical resection urging the need to identify treatments that block the invasive growth of glioma in order to completely resect the tumor and improve overall survival (118).

The GBM microenvironment consists of a wide variety of immune, stromal, and glial cells with a highly abnormal tumor vasculature that together can form distinct vascular tumor niches (113). In these niches, tumor and brain resident cells interact via cell contact and paracrine signals to promote tumor cell maintenance, growth, and protection against cancer therapies. From the rim of the tumor, tumor cells commonly infiltrate the normal brain parenchyma directly or by moving along nerves and basement membranes including those of vascular structures where they become closely associated with microglial cells (113, 117). It is believed that microglial cells are recruited to and accumulate around tumors as part of a wounding response. Under the influence of glioma cells, microglial cells acquire pro-tumorigenic properties (96). It has been shown that microglial cells can favor tumor progression by releasing pro-tumorigenic growth factors (119), boosting angiogenesis and invasion (120), and helping the tumor to escape the immune response by suppressing cytotoxic T-cell functions and inducing a T_reg_ immunosuppressive response (121).

In addition, peripheral macrophages accumulate within GBMs due to chemoattractive signals emanating from the tumor, which enable macrophage infiltration through the abnormal and leaky tumor vasculature (113). Tumor-associated macrophages (TAMs), including microglial cells and monocyte-derived macrophages, can represent up to 50% of the tumor mass in GBMs (122). While GBM-infiltrating macrophages are predominant in the tumor (Tumor-associated macrophages (TAMs)), resident microglia reside preferentially in the tumor periphery (64, 123).

**Figure 4:**
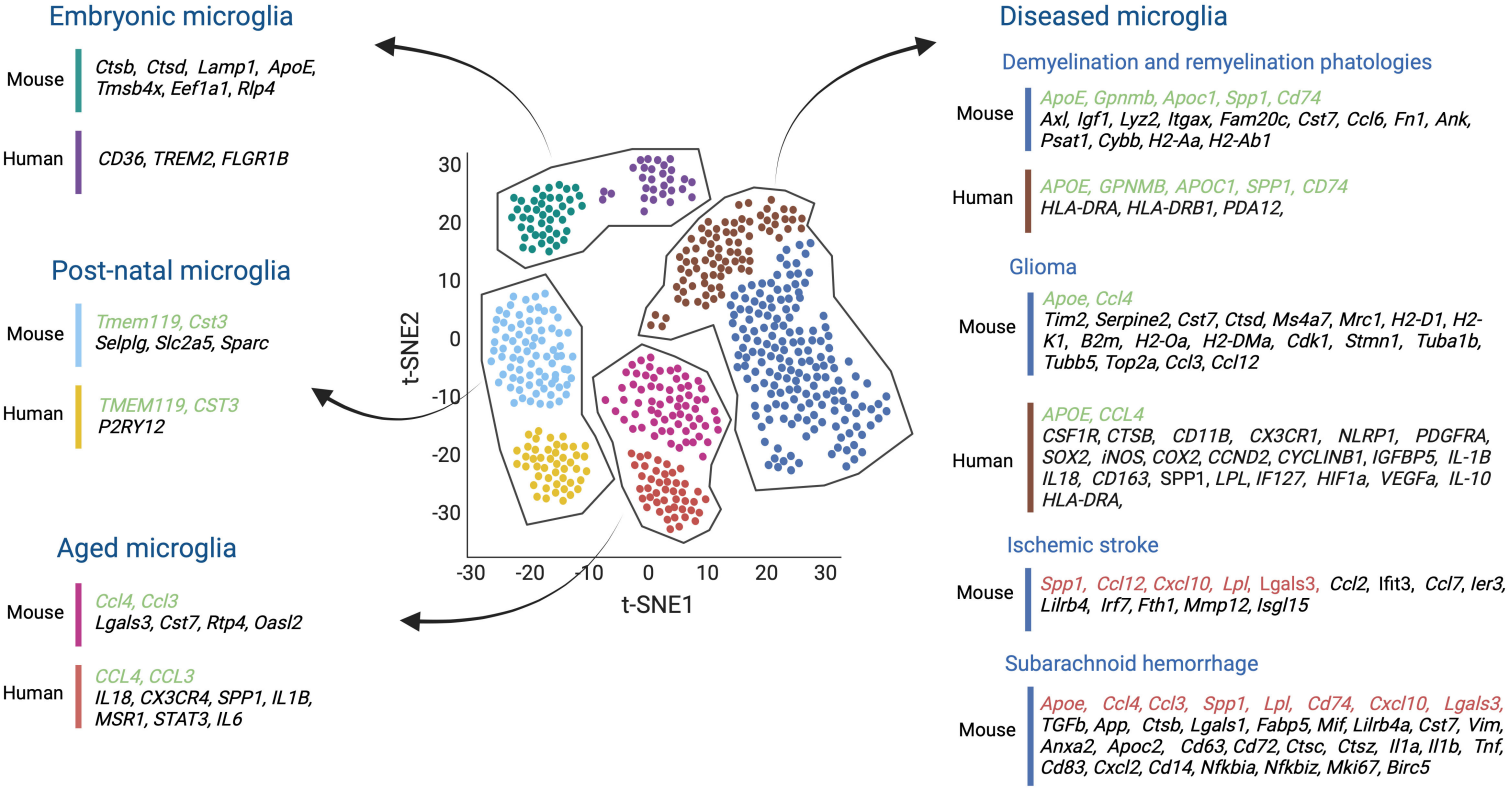
Single-cell transcriptomics studies both in mouse and human microglia, conducted across various stages of development, aging, and in the context of several brain diseases such as demyelination and remyelination pathologies, glioma, ischemic stroke, and subarachnoid hemorrhage, have shed light on the molecular diversity of microglial cells. These studies have helped us to understand the transcriptomic similarities (highlighted in green) and differences (highlighted in black) between mice and humans in healthy and diseased brains. Moreover, comparisons were made between mouse microglial gene expression during ischemic stroke and subarachnoid hemorrhage, and the transcriptional profile of other disease-associated microglia, proving insights into shared molecular responses across different contexts (highlighted in red). Created by Biorender.

Several studies have shown that although microglial cells in tumor-bearing mice change their homeostatic microglia signature, one can, to a certain extent, still distinguish glioma-activated microglia from infiltrating TAMs, in part by determining their transcription profile by scRNAseq (**Figures 1**, **4**) (124).

TAMs, like microglia, comprise a diverse population capable of both anti-inflammatory/pro-tumorigenic and pro-inflammatory/anti-tumorigenic phenotypes. Pro-inflammatory TAMs can be cytotoxic and have the ability to present antigens to recruit cytotoxic T cells to attack the tumor cells (51). Conversely, anti-inflammatory TAMs can participate in tissue remodeling and angiogenesis, while secreting anti-inflammatory cytokines such as IL-10 and TFG-β to promote tumor growth (125). It is important to note that recent studies revealed that glioma-associated microglia/macrophages display a broader profile of states reflected in their distinct expression profiles that go beyond the immune-related phenotypes. For instance, murine and human TAMs display a higher expression of *Gpnmb* and *Spp1* compared to naïve microglia or peripheral macrophages, which has been correlated with poor patient survival (126). Additionally, RNAseq analysis showed that microglia cells in close conjunction with glioma cells presented a different expression profile than those that did not interact with tumor cells. For example, glioma-associated microglial cells downregulated transcription of factors that promoted tumor sensing and killing like *Siglech*, *Cd33*, *Gpr34* (microglia sensors for GBM cell ligands), *P2ry12* and *P2ry13* (microglial ATP receptors responsible for trigger an acute inflammatory response) respectively. Moreover, they upregulated the expression of genes, elevating molecules that are involved in the degradation of the extracellular matrix (ECM), promoting tumor migration and growth (i.e. *Mmp12*, *Mmp13*, *Mmp14*), and immune suppression such as *Pd-l1* and *Pd-l2* (127). All these observations infer that TAMs are diverse cell populations that most likely play distinct roles in tumor progression and immune evasion.

Given the convincing results of microglia/macrophage implication in glioma growth and progression, targeting approaches similar to those described for microglial cells in the healthy brain, have been assessed. The colony-stimulating factor-1 (CSF1)-CSF1R signaling axis in macrophages/microglia not only regulates microglia/macrophage differentiation and survival, but it also enhances an immune-tolerant state (95, 128). Congruently, pharmacological inhibition of CSF1R (BLZ945) in a PDGFB-driven GBM mouse model skewed macrophages to an immune-stimulating state blocking glioma progression and enhancing survival without depleting microglial cells (95). However, several other preclinical and clinical studies did not observe any significant beneficial effects of CSF1Ri therapy. While recent studies in mice have confirmed that CSF1R inhibition decreased tumor growth in PDGFB-driven GBM, they also reported that it failed to show beneficial effects in mice bearing mesenchymal RAS-driven GBMs or other proneural and mesenchymal GBMs (129). Further, TAM depletion with CSF1Ri (PLX5622) did not confer any survival benefit in oligodendrocyte progenitor cell lineage-associated GBMs or subventricular zone neural stem cell-associated GBMs (130). In line with the preclinical studies, the CSF1Ri PLX3397 did not produce beneficial effects in a GBM Phase I/II clinical trial (131).

Another signaling pathway implicated in promoting protumorigenic features in macrophages has been described for PI3Kγ, a myeloid cell-specific PI3K subunit. PI3Kγ promotes immune suppression by activating mTOR-S6Kα-C/EBPβ and inhibiting NFkβ in macrophages. While PI3Kγ-mTOR-mediated immunosuppression promoted tumor growth, PI3Kγ inhibition reverses these effects by shifting macrophages towards NFkβ -dependent pro-inflammatory polarization (132–134). Moreover, a recent study has shown that PI3Kγ inhibition decreased TAM accumulation in the GBM microenvironment, enhancing temozolomide (TMZ) treatment response in orthotopic murine GBM models (119).

Microglia can also support GBM growth by becoming oxidatively stressed and impairing antigen presentation to CD8^+^ T-cells. Under these conditions, microglia upregulate the transcription factor *Nr4a2*, which alters cholesterol homeostasis. Myeloid-specific *Nr4a2* knockout reversed immunosuppression by upregulating the MHC-I complex and enhancing antigen presentation for CD8 T cells, subsequently reducing GBM growth (135).

Additionally, inhibiting HMOX1 activity in microglia reduced IL-10-produced T cell exhaustion whereas the JAK1/2 inhibitor ruxolitinib boosted T cell activation by reducing immunosuppressive macrophages (136). Moreover, targeting P-selectin (SELP), a leukocyte adhesion molecule, delayed GBM growth, prolonged survival, and improved immune cell infiltration *in vivo*. scRNAseq analysis of GBM-shSELP-derived microglia/macrophages showed an increase in pro-inflammation and T-cell activity (137). Thus, P-selectin may play a crucial role in modulating the microglia/macrophage immune response in GBMs.

These examples underscore the impact of blocking immunosuppressive key signaling nodes in macrophages, which albeit not microglia-specific, limit the overall immunosuppressive milieu in GBM. As targeting macrophages with CSF1Ri or blocking negative immune checkpoint regulators like PD1, PD-L1 or CTLA4 are not sufficient as stand-alone therapies to improve the outcome in GBM patients, combining these strategies, also in conjunction with standard chemo- and radiation therapies or other immunotherapies are highly anticipated as they may hold great potential for improving the treatment of gliomas (138).

Overall, the studies exemplified above, provide ample evidence of microglia and macrophage implications in cerebrovascular diseases, including stroke, aneurysm, and glioma. Their responses are plastic and influenced by the complex and dynamic microenvironment of the CNS in the respective conditions. Transcriptomics have greatly helped to gain more insight into the different microglia and macrophage states and their involvement in CNS disease conditions. Understanding the underlying mechanisms of microglial activation and participation in these diseases may offer opportunities for developing targeted therapies to modulate microglial and macrophage activity and improve patient outcomes.

## 4 Lessons learned from microglia transcriptomics

### 4.1 Microglial during development and in homeostasis

Advances in single-cell transcriptomic studies have provided invaluable insights into the molecular diversity of microglial cells in humans and mice at different stages of life and in several disease contexts. For instance, the transcriptional profile of different microglial populations was analyzed across several developmental stages in healthy and diseased mouse brains and compared to human MG populations. Single microglial cells from multiple brain regions of healthy embryonic E16.5, juvenile (3 weeks), and adult (16 weeks) mice were analyzed and compared to microglia isolated from mice with neurodegenerative and demyelinating conditions. Unsupervised clustering inferred thirteen different clusters corresponding to developmental microglia (C1-C10), microglia during neurodegeneration (C11), and microglia under demyelination and remyelination conditions (C12-C13). Interestingly, microglia clustered separately depending on the pathological state,’ (139).

Among the microglial developmental clusters (C1-C10), two central states were identified reminiscent of embryonic microglia (C1-C6) and postnatal microglia (C7-C10), which include juvenile and adult mouse samples. All embryonic-related microglia clusters (C1 to C6) generally exhibited the highest heterogeneity. They all expressed lysosome-related genes such as *Ctsb*, *Ctsd*, and *Lamp1*. The C1, C2, and C5 clusters displayed *Apoe*, and high levels of Tmsb4x, Eef1a1, while Rlp4 gene expression characterized the C6 microglia cluster. Interestingly, factors associated with homeostatic microglia like *Tmem119*, *Selplg*, and *Slc2a5* inferred the highest expression in the postnatal clusters (C7-C10). In addition, clusters C9 and C10 highly expressed *Cst3*, a cysteine proteinase inhibitor involved in neurodegenerative diseases of the CNS, and *Sparc* (**Figure 4**).

Microglia in both demyelination and remyelination conditions (C12-C13) increased the expression of *Apoe*, *Axl*, *Igf1*, *Lyz2*, *Itgax*, *Gpnmb*, and *Apoc1.* The C12 cluster corresponding to microglia in demyelination was enriched in *Fam20c*, *Cst7*, *Ccl6*, *Fn1*, *Ank*, *Psat1*, and *Spp1* genes. Still, the C13 remyelination cluster was characterized by high Cybb and MHC II gene expression (i.e., *Cd74*, *H2-Aa*, *H2-Ab1*) being reflective of an immune-activated state (**Figure 4**).

Finally, to compare the similarities and discrepancies between murine and human microglia cells, microglial cells were obtained from healthy human brain tissues. Unbiased clustering showed four main clusters (HHu-C1 to HHu-C4) that partially overlapped with the adult murine microglia clusters. For example, *CST3*, enriched in the mouse C9 and C10 clusters, was also upregulated in HHu-C1 and HHu-C2 clusters. Moreover, CD45^+^ cells isolated from the brains of patients with early active multiple sclerosis (MS) were analyzed and compared to the human microglia of healthy patients. Unbiased clustering revealed ten different clusters (Hu-C1 to Hu-C10), but only Hu-C2 to Hu-C8 expressed microglial core genes (i.e. *TMEM112* and *P2RY12*). Hu-C5, Hu-C6, and Hu-C7 clusters corresponded to microglia from healthy brains and expressed high levels of the MG-core genes, resembling an expression profile of healthy mouse postnatal C7 to C10 clusters. In addition, the microglial Hu-C2, Hu-C3, and Hu-C8 clusters from the diseased MS brains resembled the C12 and C13 clusters of disease-related mice. All clusters were enriched in the expression of *APOE* and downregulated the typical MG-core genes. Specifically, Hu-C2 had high *APOC1* and *GPNMB levels*, and Hu-C3, like the murine C13 cluster, exhibited increased transcription of MHC II- related genes (i.e., *CD74*, *HLA-DRA*, and *HLA-DRB1*). Hu-C8, similar to the mouse C12 cluster, showed high levels of *SPP1* and *PADI2* expression (139) (**Figure 4**).

Taken together, during homeostasis different microglia subpopulations were found from embryonic to adulthood, but they did not cluster separately and showed a somewhat similar expression profile with the human homologs. However, microglia responses in distinct disease settings (i.e., neurodegeneration, demyelination, and remyelination) were transcriptionally quite different from each other as their responses were tailored to the specific pathology.

### 4.2 Microglia during aging

Other scRNAseq studies have investigated transcriptional changes of aging microglial cells during murine and human lifespans. In a first study, microglial cells were isolated from mouse embryos at E14.5, from postnatal brains at P4/5, from brains of late juvenile mice at P30, P100, and aged mice at P540. Aged microglia were found to express inflammatory-related genes such as *Lgals3*, *Cst7*, *Ccl4*, *Ccl3*, *Rtp4*, and *Oasl2*, and revealed a limited transcriptomics heterogeneity compared to microglia from younger mice (140) (**Figure 4**). These results were supported by another study which demonstrated that early postnatal microglia were more heterogeneous than adult microglia. They also discovered an early postnatal phagocytic state of proliferative-region-associated microglia (PAM) with a similar gene signature to that of degenerative disease-associated microglia (DAM) (141). Interestingly, scRNAseq on human microglia isolated from pre-natal (2nd trimester), pediatric (18 months – 24 months), adolescent (10-14 years old), and adult (40-62 years old) brain samples revealed pre-natal microglia with a higher phagocytic activity (i.e., upregulation of *CD36*, *TREM2*, and *FCGR1B*) compared to those at an adult stage. Adult microglia, however, were more immune responsive (i.e., upregulation of the pro-inflammatory cytokines *IL18* and *CX3CR4*, the interferon-response genes *CCL3* and *CCL4 as well as SPP1*, *IL1B*, and *MSR1*) (142) (**Figure 4**). Likewise, microglia from patients aged 50 years and older had higher *SPP1* expression levels compared to microglia from younger adults (143) (**Figure 4**). Further support for an increased microglial inflammation state during aging stems from Lopes et al., exposing upregulated inflammatory pathway activities in microglia of humans with increasing age, including *STAT3* and *IL-6* signaling, the (LXR)/retinoid X receptor (RXR) activation and liver X receptor (144) (**Figure 4**).

Taken together, scRNA-seq analyses revealed reduced transcript heterogeneity but increased inflammatory signatures in aging microglial cells, both in mice and humans.

It is noteworthy that these studies would further gain from revealing potential gender differences in microglia expression profiles and subsequent function as several studies already described differences in microglia among genders from several developmental states (i.e. embryonic, early postnatal, adult, and aged) and neurodegenerative diseases. For example, in a model of ischemic stroke, transplanting female microglia into male mice reduced the progression of ischemic damage compared to transplanting male microglia, suggesting a protective role of female microglia in ischemia (145, 146).

### 4.3 Microglial transcriptomics in CNS disease

#### 4.3.1 Glioma

A recent study performed scRNA-seq analysis of murine GL261 tumors and discovered two new microglia states compared with microglia from naive brains. Both states showed an upregulation of *Timp2*, *Serpine2*, *Cst7*, and *Ctsd*, genes related to the reorganization of the extracellular matrix, and *Apoe*, *Ms4a7*, and *Mrc1*. However, while one of these GBM-related microglia states was enriched in transcripts for MHC I (i.e., H2-D1, H2-K1, and B2m) and MHC II (i.e., *H2-Oa* and *H2-DMa*), the other one mainly expressed genes related to cell proliferation, including *Cdk1*, *Stmn1*, *Tuba1b*, *Tubb5*, and *Top2a*. Transcriptional network analysis further demonstrated that MHC II^high^-activated microglia express more chemokine-encoding genes (i.e., *Ccl3*, *Ccl4*, and *Ccl12*), suggesting that this subset of microglial cells may help to recruit other immune cells (124) (**Figure 4**).

Similarly, scRNA-seq analysis of human microglia from five glioma patients also identified a subset of high-grade glioma-associated microglial cells (HGG-AM) in IDH1-WT/SETD2-mutant GBM. This specific microglia subset expressed core microglia markers, including *CSF1R*, *CTSB*, and *CD11B.* It also upregulated CX3CR1, NLRP1, PDGFRA, and SOX2, while decreasing the expression of several homeostatic genes, including *P2RY12* and *TMEM119.* Interestingly, this cluster exhibited pro-inflammatory and proliferative signatures (i.e., *NLRP1*, *iNOS*, *COX2*, *CCND2*, *CYCLINB1*, *IGFBP5*) and shaped the cytokine microenvironment (i.e., *IL-1B* and *IL18*) as previously reported for the murine glioma-associated microglial cells (147) (**Figure 4**). In line, Sankowski et al., identified two human glioma-associated microglia (GAMs) clusters with low expression of the microglia core genes, including *CX3CR1* and *SELPLG*, and high expression of metabolic, inflammatory, and interferon-associated genes such as *CD163*, SPP1, *APOE*, *LPL*, and *IF127*. In addition, these clusters presented a MHC II gene signature similar to glioma-associated microglia cells in mice. Some cells also expressed hypoxia-associated genes such as *HIF1a* and *VEGF-A* (143) (**Figure 4**). Moreover, another single-cell study revealed immunosuppressive CD163^+^HMOX1^+^ microglia in human GBM samples that expressed tumor-associated macrophage genes such as *CD163*, *CCL4*, *APOE*, and *HLA-DRA* and showed a significant enrichment of pathways involved in antigen-processing and cytokine signaling similar to the mouse glioma-associated microglial cells. Additionally, this population induced T-cell exhaustion via the release of IL-10 (136) (**Figure 4**).

Taken together, murine and human glioma-associated microglia cells exhibit a similar pro-inflammatory and proliferative phenotype with a characteristic upregulation of several metabolic pathways and reduced expression of microglia core genes.

#### 4.3.2 Stroke

scRNAseq analysis of brain areas with an ischemic stroke helped to identify unique microglia subpopulations with specific transcriptional profiles. For example, Zheng et al., performed scRNAseq on the MCAO (middle cerebral artery occlusion) mouse model of ischemic stroke and identified five unique microglia states (MG 0-4) (148). All microglia clusters generally expressed the typical microglial markers (i.e., *Gpr3*4, *Olfml3*, *P2ry12*, *TMEM119*, *Selplg*, and *Siglech*) (**Figure 4**). However, most ischemic-associated microglia clusters (MG 2-4) had a relatively low expression of these markers and contained, to some extent, inflammatory signatures. For instance, these microglia upregulated several monocyte chemotactic protein family genes such as *CCL12*, *CCL2*, and *CCL7*, as well as *IER3* (a gene associated with hypoxia). In addition, typical genes of neurodegeneration-associated microglial subgroups (i.e., *Lgals3*, *Lilrb4*, *Lpl*, *Spp1*, and *Fth1*) (8) and *MMP12* (matrix metalloproteinases 12) known to cause damage to the BBB upon ischemic stroke (149) were also upregulated (**Figure 4**). Moreover, the expression levels of genes involved in IFN signaling, such as *Isgl15*, *Ifit3, Irf7*, and *Cxcl10*, were also increased. Additionally, transcription of genes implemented in cell proliferation like *Top2a*, *Stmn1*, *Birc5*, and *Ube2c* were also upregulated (148) (**Figure 4**).

#### 4.3.3 Subarachnoid hemorrhage

A scRNAseq study of microglia post subarachnoid hemorrhage (SAH) compared to healthy mouse brain microglia identified three clusters in the SAH-affected brains (SMG-C5, SMG-C6, and SMG-C7), with all exhibiting inflammatory signatures accompanied by high expression of *CCL*, *GALECTIN*, *TGFb*, *APP*, and *SPP1*. The SMG-C5 cluster revealed high expression of *Spp1*, *Lpl*, *Apoe*, *Ctsb*, *Lgals1*, *Lgals3*, *Fabp5*, *Mif*, *Lilrb4a*, *Cst7*, and *Vim* (**Figure 4**). Noticeably, some of these genes were upregulated in disease-associated microglia (DAM) and injury-responsive microglia (IRM). In addition, a gene expression profile entailing *Anxa2*, *Apoc2*, *CD63*, *CD72*, *Ctsc*, and *Ctsz* was also upregulated and similar to that of microglia in developing brain and in mouse models of Alzheimer’s disease and lysolecithin (LCP) injury (140, 150). The SMG-C6 cluster expressed several cytokines (i.e. *Il1a*, *Il1b, Tnf*, *Ccl4*, and *Ccl3*), chemokines (i.e. *Cxcl10* and *Cxcl2*), and other immune signal-regulating genes (i.e. *CD83*, *CD74*, *CD14*, *Nfkbia*, and *Nfkbiz*). Some of these markers were previously identified in IRM subpopulations in Alzheimer’s disease and multiple sclerosis (MS) (140, 150, 151) (**Figure 4**). The SMG-C7 cluster increased the transcription of genes promoting proliferation, such as *Birc5*, *Mki67*, and *Fabp,5* like in developing microglia (152) (**Figure 4**).

In summary, single-cell studies have provided an excellent overview of the distinct microglial transcriptomic profiles in neurovascular diseases such as glioma, ischemic stroke, and subarachnoid hemorrhage. Independently of the number of microglia clusters found, some presented similarities. For instance, the disease-associated microglia (GAMs in glioma, ischemic-associated MG in MCAO, and DAM and IRM in SAH exhibit a decrease in several microglial core genes such as CX3CR1 and TMEM119, a high presence of inflammatory signatures, and an upregulation of distinct proliferation markers. These findings underscore the dynamic nature of microglial responses in diverse neurological conditions, providing valuable insights for potential therapeutic interventions targeting these intricate immune cells.

## 5 Microglia targeting for treating cerebrovascular diseases

### 5.1 Microglia depletion in brain disease

In the context of a stroke, researchers have explored microglial depletion through various approaches to better understand its impact on disease outcomes. For instance, pharmacological agents such as PLX5622 and PLX3397 have been used to deplete microglia before middle cerebral artery occlusion. Surprisingly, results indicated that microglial depletion worsened disease outcomes, leading to increased brain injury and altered neuronal activity. Thus, it is conceivable that, at an early stage, microglia may still be able to elicit protective functions against injury (153, 154). Similarly, experiments involving microglia depletion using PLX3397 and CX3CR1^CreER^ iDTR transgenic mice, both before and after middle cerebral artery occlusion, demonstrated aggravated neurological functions, brain inflammation, cell death, and leukocyte infiltration within the initial days following stroke (66, 155, 156). These findings confirm that microglial cells provide beneficial effects during the first days after stroke, reducing cell death, neurological deficits, leukocyte infiltration, and neuroinflammatory markers (66, 155, 156). However, long-term microglia depletion using PLX5622 post-ischemic stroke decreased neuroinflammation, affecting the delicate homeostatic balance (157). Currently, the optimal window for microglia depletion to enhance recovery remains unknown. Emerging data suggest that microglial activity and function are essential both before and within the initial days of stroke to reduce peripheral cell infiltration and cytotoxicity. Nevertheless, prolonged depletion may become detrimental (158), highlighting the need to target microglia within an optimal therapeutic window.

In summary, MGs exert both beneficial and detrimental effects in the context of a stroke. Their presence during the early onset of a stroke appears to have a neuroprotective role, while long-term depletion may disrupt the delicate balance of neuroinflammatory responses. Understanding the precise timing and duration of microglial interventions may pave the way for targeted therapeutic strategies to enhance stroke recovery and improve patient outcomes.

In the setting of aneurysms, microglia play a crucial role in the inflammatory response within the surrounding area, which may contribute to secondary brain injuries, perpetuating the cycle of inflammation after an initial injury (91, 109). Therefore, several studies have focused on the depletion of MGs via genetic and pharmacological approaches to study its impact on the disease’s development and its effect on the recovery post-aneurysm. For instance, studies have reported that microglia depletion *in vivo* using the CD11b-HSVTK mouse model, in which all CD11b^+^ cells are depleted upon ganciclovir administration in the brain, resulted in reduced neuronal cell death and improved vasospasm following aneurysmal subarachnoid hemorrhage (SAH) (108). Similar results were observed using liposome-encapsulating clodronate. Upon intracerebroventricular injection of clodronate in early-phase SAH, a decrease in neuronal cell death was observed, suggesting that neuronal apoptosis was microglial-dependent. However, in late-phase SAH, neuronal cell death was associated with TLR4- toll receptor-associated activator of interferon (TRIF) and was independent of the microglia presence. In both early and late-phase SAH, microglia were shown to be necessary and sufficient to cause vasospasm since microglia depletion improved the vasospasm outcome (159). In addition, MG depletion with the CSF1R inhibitor PLX3397 following SAH reduced neuronal cell death due to a decrease in microglia accumulation and activation (160).

These findings highlight the importance of microglial cells in the pathophysiology of aneurysmal SAH and suggest that strategies to modulate microglial activation could mitigate secondary brain injuries and vasospasms in individuals with aneurysms. Further research may provide valuable insights into novel treatment strategies for this serious medical condition.

Microglia/TAM-targeting therapies have also been conducted in gliomas, given their role in promoting glioma progression, angiogenesis, and invasion (120). Several studies have indicated that targeting MGs via CSF1R inhibition can delay recurrence and extend overall survival in several glioma models by inducing a more pro-inflammatory phenotype in immune cells (95, 161). Despite these promising results, CSF1R inhibition has not demonstrated efficacy in pre-clinical and clinical glioma studies (131, 162, 163). Several reasons have been identified for this lack of success. CSF1R inhibition monotherapy may not be sufficient to switch the balance of TAMs/microglia to an immunostimulating state and it can promote the development of acquired resistance mechanisms such as 1) increased insulin-like growth factor-1 receptor (IGF-1R) expression and aberrant phosphoinositide 3-kinase (PI3K) activity on cancer cells (162), 2) enhanced influx of Foxp3+ regulatory T cells (Tregs) (164), 3), high recruitment of pro-tumorigenic cell populations such as myeloid-derived suppressor cells (165), 4) elevated levels of glioma-secreted factors such as granulocyte-macrophage CSF (GM-CSF) and interferon-y (IFN-y) (95), and 5) up-regulation of T-cell immune checkpoint molecules such as programmed cell death 1 ligand (PD-L1) and cytotoxic T-lymphocyte-associated protein 4 (CTLA-4) (166, 167), increased recruitment of tumor-infiltrating lymphocytes (TILs) (168).

Due to the failure of monotherapy using CSF1R inhibitors, researchers are currently exploring combinatorial therapies as a promising approach to target the tumor from different angles. For instance, in the context of gliomas, the combination of CSF1R inhibitors and radiotherapy has shown synergistic results by effectively suppressing tumor growth (169). Likewise, when CSF1R inhibitors were combined with anti-PD-1 therapy, there was a notable increase in overall survival in mice (161, 168). In addition, CSF1R inhibition was also combined with anti-angiogenic therapy using cediranib (a VEGFR2 inhibitor), reducing cell proliferation and altering the tumor morphology (129). Most interesting, a new phase I study using SYHA1813, a VEGFR and CSF1R dual inhibitor, has been reported to show anti-tumor activity in patients with recurrent High-Grade Gliomas and Advanced Solid Tumors (170).

Further research is needed to explore combination therapies and strategies that can potentiate the effects of CSF1R inhibition while addressing the mechanisms of acquired resistance. Understanding the complex interplay between microglial cells and the tumor microenvironment will be critical in developing effective treatments for glioma.

### 5.2 MG repopulation in brain disease

To date, there are no reported studies on the outcomes of microglial repopulation upon depletion in stroke, aneurysm, or glioma. Further research in this area is essential to understand the dynamics of microglial repopulation and its potential implications on the recovery of these diseases upon previous microglia depletion. However, one can speculate about the potential positive effects of repopulating microglia. For instance, in the context of stroke, repopulated microglia that elicit anti-inflammatory properties could help decrease the inflammation in the injured area, facilitating a faster recovery. This, however, is challenging as it would require stable reprogramming of repopulating microglia to hinder their polarization to a pro-inflammatory state in the brain, which would exacerbate the stroke-induced injury (45, 90).

Similarly, in the context of aneurysms, the phenotypic state of the newly re-populated microglia will be essential for the outcomes of the inflammatory responses that will affect the stability of the aneurysm wall and the resolution of the injury upon aneurysm disruption. Since anti-inflammatory macrophages have been shown to secrete several cytokines and chemokines, the best possible outcome would be that repopulating microglia adopt a wound-healing state to facilitate the resolution of the cerebral injury and hemorrhage upon aneurysm disruption (91). However, the same concern as in stroke arises if re-populating microglia become pro-inflammatory and perpetuate inflammation (91).

In the situation of gliomas, repopulating microglia should be immune-stimulating to enable restoration of immune surveillance and anti-tumor immune responses. This would also facilitate the recruitment of other immune cells, such as T cells, natural killer (NK) cells, and dendritic cells, thereby contributing to a more effective anti-tumor immune response (171). However, it is essential to consider the potential negative effects as well. Re-populating microglia may promote immunosuppression, angiogenesis, and tissue remodeling, creating a tumor-supportive environment (96, 113, 172). Additionally, in some cases, re-populated microglia may contribute to the suppression of anti-tumor immunity by inducing the recruitment of regulatory T cells (Tregs) or myeloid-derived suppressor cells (MDSCs), which can dampen the immune response (121, 171).

Expanding our knowledge in this field will help to decipher whether microglia-targeted therapies could be a good treatment option for these devastating diseases. Further research is needed to ensure that MG-targeting or depletion strategies do not back-fire and improve the patient’s prognosis and quality of life.

### 5.3 Microglia re-education or re-programming in brain disease

An alternative strategy that circumvents the need for microglia depletion and repopulation is an endogenous re-education of microglia/macrophages.

Microglia re-programming has long been studied in the context of gliomas given the key role that microglia/macrophages play in this disease progression. Some studies have shown that CSF1R inhibition with BLZ945 and PLX3397 depleted macrophages in healthy tissues. However, glioma-TAMs were not depleted with BLZ945 but reprogrammed toward a less pro-tumoral phenotype (95, 173). Why certain CSF1R inhibitors deplete microglia while others reeducate microglia and TAMs, is unknown. As it may be, CSF1R inhibitors have only shown modest improvement in the short-term survival of mice bearing PDGF-B driven gliomas (162). Thus, other strategies have been used to re-educate TAMs, including the inhibition of the myeloid PI3K pathway, thereby shifting macrophage polarization toward a pro-inflammatory phenotype in preclinical studies of GBM (119) (**Figure 3**).

Another method to re-program TAMs is via blocking the CCL5/CCR5 signaling either by targeting CCR5 (a G-protein coupled receptor) or by using a neutralizing antibody against CCL5. CCL5 and CCR5 expression correlates with poor prognosis and shorter patient survival in GBMs. It has been shown that glioma invasion depends on the CCL5/CCR5 signaling axis and it is inhibited by the CCR5 antagonist maraviroc (174). Moreover, CCR5 is also expressed in microglia/macrophages and has been linked to inducing their activation and polarization mediating immunosuppression in glioma (174, 175). Maraviroc treatment was able to shift TAMs to become more anti-tumoral by decreasing the gene expression of *Arg1* and *IL-10*, and increasing the levels of NO and *IL-1β* upon microglia/macrophages LPS-IFNγ-conditioned medium stimulation *in vitro* (175) (**Figure 3**).

CD40 agonist treatment in combination with D2C7-IT, a dual immunotoxin that targets specifically the human epidermal growth factor receptor (EGFR) and mutant EGFR variant III (vIIII) proteins, has also been reported to repolarize macrophages and boost their antitumoral immune response in GBM models increasing patient survival outcomes (176) (**Figure 3**). Interestingly, a phase I trial with D2C7-IT in combination with 2141-V11 (an anti-CD40 monoclonal antibody) has recently started in patients with recurrent malignant glioma (NCT04547777).

Moreover, engineered oncolytic viruses specifically designed to kill cancer cells have shown a significant impact on GBM progression since these viral particles expressing IL-12 increased tumor cell death and shifted the TAMs towards a pro-inflammatory phenotype, improving anti-PD1 and anti-CTLA-4 immunotherapies outcomes (177) (**Figure 3**). Additionally, certain studies also showed that dual-inhibition of VEGF and Ang2 in glioma models prolonged survival compared with single VEGF inhibition by reprograming Tie2^+^ TAMs toward a pro-inflammatory/anti-tumoral phenotype (178) (**Figure 3**).

In summary, microglia/TAM re-education offers a promising alternative strategy to overcome any obstacles that may appear upon their depletion and consequent re-population. Several approaches of re-educating microglia/TAMs have provided promising effects on reducing tumor growth in pre-clinical studies, but the effects in GBM remain modest. Thus, TAM/microglia repolarization toward a more pro-inflammatory/anti-tumoral phenotype will be more beneficial in combination with other immune-related therapies such as anti-PD1, anti-CTLA-4, or CAR-T, or even just standard chemo-or radiation therapy improving tumor growth reduction and overall patient survival.

### 5.4 Myeloid adoptive cell transfer

Another option is to re-populate microglial cells via adoptive cell transfer of pre-defined myeloid cells. Functional blood-derived monocytes, bone marrow-derived macrophages, or stem cell-derived myeloid cells could be used to re-populate the microglia-depleted brain in different stages, including pre-activated or genetically modified forms (**Figure 3**). It has been demonstrated that blood monocytes can be differentiated into blood monocyte-derived microglial cells via treatment with GM-CSF and IL-34. These cells share striking similarities with the brain-resident microglia, exhibiting a ramified morphology and expressing microglial genes such as *Tmem119* and *P2ry12*. Moreover, they display efficient phagocytic capabilities, contributing to debris clearance. Interestingly, these microglia-like cells display distinct characteristics from monocytes, macrophages, and dendritic cells (179, 180).

A different approach for myeloid adoptive cell transfer would be the generation of microglia-like cells from bone marrow-derived macrophages. For instance, it has been reported that bone marrow-derived mononuclear cells treated with GM-CSF and IL-4 became microglia-like cells, demonstrating therapeutic effects in a mouse model of amyotrophic lateral sclerosis (181) (**Figure 3**).

Another more recent approach is the generation of microglia-like cells from human inducible pluripotent stem cells (hiPSCs). For example, it has been shown that CSF1 and IL-34 can differentiate hiPSCs into Tmem119^+^/P2RY12^+^ microglia-like cells (182). Furthermore, complementation with specific factors and co-culture with astrocytes have also been successful in converting hiPSCs into microglia-like cells (183) (**Figure 3**).

All the above-mentioned options rely on the differentiation of “precursor cells” into functional microglial-like cells. The newly derived MGs could be transplanted in cases where the existing MGs/macrophages are depleted, effectively restoring functional macrophages aiming at the resolution of cerebrovascular diseases. Furthermore, these MG-like cells could be stimulated *in vitro* prior to cell transfer, enhancing their ability to resolve injuries or fight against the tumor cells in the context of GBMs.

Another unexplored alternative involves the transplantation of fully functional microglial cells into patients (**Figure 3**). This cell-based therapy has already been studied in the context of stroke, aiming at attenuating brain injury and improving neurological outcomes. For instance, transplantation of HMO6 cells (a human microglia cell line) into rats that had chronic cerebral ischemia resulted in a reduction in white matter damage caused by ischemia, attributed to the downregulation of matrix metalloproteinase (MMP)-2 levels in microglia (184). Moreover, other studies explored the transplantation of M2-like activated murine microglia cell line BV2 into recipient mice post-middle cerebral artery occlusion (MCAO). This intervention led to a decrease in brain damage resulting from ischemia and the promotion of angiogenesis (185).

As such, microglial cell transplantation has emerged as a novel therapeutic strategy to reduce the impact of stroke injuries and to improve neurological recovery upon such injuries. However, it is essential to note that further research is also required in this area which is still in its infancy. This is particularly important because some studies revealed that microglial transplantation in rats did not confer any beneficial effects in cases of permanent cerebral ischemia (186).

Overall, the potential of reprogramming and repopulating microglia through adoptive myeloid cell therapy holds significant promise for enhancing outcomes in both cerebrovascular diseases and neurological disorders. Exploiting the plasticity of these immune cells and directing them toward beneficial phenotypes could open up novel therapeutic pathways.

## 6 Conclusions and future perspectives

In recent years, significant progress has been made in our understanding of microglial functions in maintaining homeostasis in the healthy brain, and their implication in neuroinflammatory diseases. Thereby, a major advancement has been the development of single-cell and multi-omics technologies and the integration of gene and protein expression profiles, which have identified diverse and context-dependent microglial states across species and models. These analyses have provided new insight into the molecular mechanisms of microglia functions, revealing multiple cell states reflective of their plasticity to display their context-dependent multifunctionality during development, homeostasis, and CNS disease. These studies also underline the recent request for a new microglial terminology to more faithfully describe microglia’s dynamic states and depict their complex nature and function in a context-dependent manner (47).

Notwithstanding, while researchers have made substantial contributions regarding the origin, morphology, and states of microglial cells in healthy conditions, it is still challenging to distinguish microglia from infiltrating macrophages in several disease settings, and, importantly, to specifically target microglia without affecting other macrophage populations. Such a distinction is crucial to infer to which extent and by which means the regulation of the neuroinflammatory response and tissue repair is commonly or distinctly orchestrated by microglial cells or other macrophage types. So far, current therapeutic strategies aimed at harnessing the neuroprotective potential or decreasing harmful responses of microglia cells affecting also other macrophages. To specifically modulate microglial behavior, there is an urgent need to gain more insight into the complex function of and interplay between microglia, other brain-resident macrophages, and infiltrating macrophages, and their communication with other cell types in the brain.

So far, targeting both microglia and macrophages has already shown some promising results in several pre-clinical settings of stroke, aneurysm, and glioma. However, as most of these approaches are not sufficient yet to mount a clinically relevant response, further research in this field is urgently needed to improve patient outcome. This may be achieved by current approaches that include combinatorial strategies with other standard or targeted treatment modalities and the right timing and duration to target microglial cells. An important asset for these strategies is also the application of myeloid adoptive cell transfers, which introduces new treatment opportunities by exploiting the surveillance and sentinel functions of microglial cells. Although still in its infancy, such an approach may hold grand promise in harnessing the patrolling capabilities inherent in microglial cells and directing them toward injury sites to facilitate prompt and targeted therapeutic interventions.

Finally, there are also new exciting developments on the horizon of which one is the potential involvement of microglial cells in the gut-brain axis. There is increasing evidence of the bidirectional communication between the gut and brain in the maintenance of physiologic homeostasis, as well as in the onset and development of several pathologic conditions, including neurodegenerative disorders, such as Parkinson’s disease and Alzheimer’s disease. Patients with neurodegenerative diseases often exhibit gastrointestinal disorders and those with inflammatory bowel diseases can experience neurological disorders. In these patients, for example, microglial cells appear to activate astrocytes (reactive astrocytes) that are implicated in perpetuating neuroinflammatory processes that contribute to apoptosis of oligodendrocytes and synaptic nerve degeneration (187, 188).

Further research in these exciting research arenas is needed to deepen our understanding not only of the microenvironmental but also of the macroenvironmental influences on microglial behavior and biology and pave the way for new and innovative treatments that will enhance brain health and combat neurological disorders.

## Author contributions

CP-M: Writing – review & editing, Writing – original draft. GB: Writing – review & editing, Writing – original draft.
